# HRPDviewer: human ribosome profiling data viewer

**DOI:** 10.1093/database/bay074

**Published:** 2018-07-11

**Authors:** Wei-Sheng Wu, Yu-Xuan Jiang, Jer-Wei Chang, Yu-Han Chu, Yi-Hao Chiu, Yi-Hong Tsao, Torbjörn E M Nordling, Yan-Yuan Tseng, Joseph T Tseng

**Affiliations:** 1 Department of Electrical Engineering, National Cheng Kung University, No.1, University Road, Tainan City, Taiwan; 2 Department of Biotechnology and Bioindustry Sciences, National Cheng Kung University, No.1, University Road, Tainan City, Taiwan; 3 Department of Mechanical Engineering, National Cheng Kung University, No.1, University Road, Tainan City, Taiwan; 4 Center for Molecular Medicine and Genetics, Wayne State University School of Medicine, Detroit, MI, USA

## Abstract

Translational regulation plays an important role in protein synthesis. Dysregulation of translation causes abnormal cell physiology and leads to diseases such as inflammatory disorders and cancers. An emerging technique, called ribosome profiling (ribo-seq), was developed to capture a snapshot of translation. It is based on deep sequencing of ribosome-protected mRNA fragments. A lot of ribo-seq data have been generated in various studies, so databases are needed for depositing and visualizing the published ribo-seq data. Nowadays, GWIPS-viz, RPFdb and TranslatomeDB are the three largest databases developed for this purpose. However, two challenges remain to be addressed. First, GWIPS-viz and RPFdb databases align the published ribo-seq data to the genome. Since ribo-seq data aim to reveal the actively translated mRNA transcripts, there are advantages of aligning ribo-req data to the transcriptome over the genome. Second, TranslatomeDB does not provide any visualization and the other two databases only provide visualization of the ribo-seq data around a specific genomic location, while simultaneous visualization of the ribo-seq data on multiple mRNA transcripts produced from the same gene or different genes is desired. To address these two challenges, we developed the Human Ribosome Profiling Data viewer (HRPDviewer). HRPDviewer (i) contains 610 published human ribo-seq datasets from Gene Expression Omnibus, (ii) aligns the ribo-seq data to the transcriptome and (iii) provides visualization of the ribo-seq data on the selected mRNA transcripts. Using HRPDviewer, researchers can compare the ribosome binding patterns of multiple mRNA transcripts from the same gene or different genes to gain an accurate understanding of protein synthesis in human cells. We believe that HRPDviewer is a useful resource for researchers to study translational regulation in human.

Database URL: http://cosbi4.ee.ncku.edu.tw/HRPDviewer/ or http://cosbi5.ee.ncku.edu.tw/HRPDviewer/

## Introduction

How cells differ in gene and protein expression and how these differences affect cellular functions are fundamental questions in biology. Traditionally, the research on the regulatory mechanism of eukaryotic gene expression was focused on the transcriptional control. Researchers assumed that the change in mRNA expression level reflects the change in protein expression level and correlates with the physiological function. Recent studies indicated that the post-transcriptional regulation of gene expression (e.g. mRNA turnover, mRNA transport and translational control) also plays an important role in modulating the protein expression level (1–3). Therefore, ‘the correlation between mRNA and protein expression’ became an issue of interest. Gygi *et al.* analyzed the mRNA and protein abundance in yeast and found that mRNA levels cannot accurately predict protein levels (4). After that, due to the development of mass spectrometry techniques which made large-scale measurement of expressed cellular proteins possible (5), more and more studies tried to address this issue in mammalian cell lines under different conditions and found moderate or even poor correlation between mRNAs and their protein products (6–8). These findings indicate that the translational regulation may have more profound impact on gene expression than originally expected. Translational regulation of mRNAs enables rapid changes in protein concentrations to maintain cell homeostasis and modulate cell physiology (9). Dysfunction or dysregulation of the translational machinery leads to human diseases such as cancer, tissue hypertrophy, neurodegeneration and inflammation (10,11).

An emerging technique called ribosome profiling (ribo-seq) is a powerful tool to study translational regulation (12). Ribo-seq can capture a snapshot of actively translated mRNAs to provide genome-wide information on protein synthesis *in vivo*. It is based on the following rationale: only actively translated mRNA fragments within a ribosome survive exposure to nucleases. Deep sequencing of these ribosome-protected mRNA fragments thus reveals the mRNAs which are actively translated by ribosomes. Besides, by using appropriate inhibitors to block ribosome movement, ribo-seq can also be used to identify the translation start sites and the speed of translating ribosomes (13,14). Moreover, ribo-seq revealed many different ribosome binding sites in the 5′ UTR under different cellular physiological conditions. These results indicate the complicated translational regulation, modulating the protein synthesis (13,15).

Nowadays, a lot of ribo-seq data have been generated in various studies. Many tools have also been developed to process users’ own ribo-seq data (16). For example, using RiboGalaxy (17), users without bioinformatics expertise can check the quality of their ribo-seq data, align their ribo-seq data to the genome or the transcriptome and visualize the results. Using RiboTools (18), users can perform qualitative analysis (e.g. identification of translational ambiguities and stop codon readthrough events) on their ribo-seq data. Using PROTEOFORMER (19), users can identify translation initiation sites (TISs) and generate a ribo-seq derived translation product database from their ribo-seq data. A description of other tools [e.g. SORFs.org (20), MTDR (21), SPECtre (22), TISdb (23), RiboDiff (24) and PUNCH-P (25)] can be seen in the review paper (16).

On the other hand, several databases have been constructed to process and store published ribo-seq data (16). Among them, GWIPS-viz (26), PFdb (27) and TranslatomeDB (28) are the three largest ones. GWIPS-viz was the first database to deposit and visualize [using UCSC genome browser (29)] published ribo-seq data from 134 studies in several model organisms (e.g. human, mouse and yeast). RPFdb was constructed to deposit and visualize [using JBrowse (30)] published ribo-seq data from 45 studies in 8 species. RPFdb also provides a list of the most translated genes for each published ribo-seq data. TranslatomeDB (28) collected 2435 published ribo-seq data in 13 species and provided the translation ratio, elongation velocity index and translational efficiency of each published ribo-seq data.

Although GWIPS-viz, RFPdb and TranslatomeDB are useful for depositing published ribo-seq data, two challenges remain to be addressed. First, GWIPS-viz and RFPdb align the published ribo-seq data to the genome and provide genome browsers to visualize the data. Since ribo-seq data aim to reveal the actively translated mRNA transcripts, aligning the ribo-seq data to the transcriptome (i.e. all mRNA transcripts in a cell) should be more biologically meaningful than aligning the ribo-seq data to the genome. Second, TranslatomeDB does not provide any visualization and the other two databases only visualize the ribo-seq data around a specific genomic location. Neither of them can simultaneously visualize and compare the ribo-seq data on different mRNA transcripts which may come from the same gene or different genes. To address these two challenges, we developed a database called Human Ribosome Profiling Data viewer (HRPDviewer). HRPDviewer contains 610 published ribo-seq datasets from 64 studies in human. Each ribo-seq dataset is aligned to the transcriptome. Users can compare and visualize the ribo-seq data mapped on different mRNAs under different physiological conditions. This kind of visualization provides novel biological insights. By viewing the ribo-seq data mapped on the mRNAs of different genes, users can know which genes’ mRNAs are highly translated under a specific physiological condition. By viewing the ribo-seq data mapped on different mRNA isoforms of the same gene, users can know which mRNA isoforms are highly translated under a specific physiological condition. We believe that HRPDviewer is a useful resource for studying translational regulation in human.

## Construction and contents

### Human ribosome profiling data collection

A total of 610 human ribosome profiling datasets (RPDs) from 64 studies were collected from Gene Expression Omnibus (GEO) database. We searched for ribo-seq datasets in GEO using several keywords (e.g. ribo-seq and ribosome profiling) and then manually checked each search result to see if we can find any ribo-seq dataset. We tried to collect as many published ribo-seq data as possible. However, we did not include any low quality ribo-seq dataset which has <2 million mapped reads. Moreover, we did not include the ribosome profiling samples treated with the translation inhibitor lactimidomycin (LTM) and harringtonine (Harr). When the samples are treated with these inhibitors, the ribosome complex will accumulate at the TIS and no complex will be found in the coding region. To prevent the misinterpretation of the calculated translational levels, we did not include this kind of ribo-seq data (e.g. GSE94460).

We assigned these 610 ribo-seq datasets to 14 research topics (Table 1). The details of these 610 collected human RPDs can be found in the download page of HRPDviewer.

**Table 1.** The 610 collected ribo-seq datasets were assigned to 14 research topics

Research topic	# of ribo-seq datasets	# of publications
Apoptosis	6	1
Cancer mechanism	85	6
Cell cycle	27	4
Circadian rhythms	48	1
Disease	62	4
microRNA regulatory effect	24	3
Mitochondrial translation	4	1
mRNA modification	34	3
mTOR pathway	62	3
Protein stability	25	2
RPF methodology	20	8
Stress condition	139	8
Translational regulation mechanism	177	22
Virus infection	106	6

Note that the number of data in Table 1 sums up to 819 not 610 is because the same ribo-seq data may be assigned to multiple topics. For example, SRR2052911 (6 h infection cytosol ribosome profiling Rep1) belongs to two topics: virus infection and stress condition.

### Data processing

**Figure 1.**
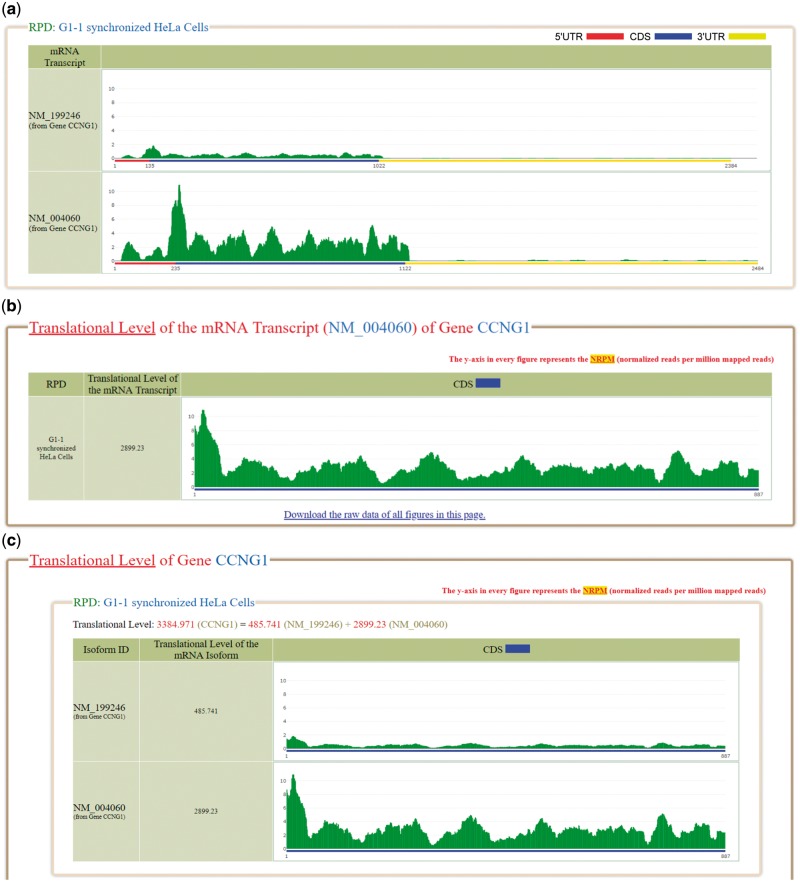
The ribosome binding patterns on the selected mRNA transcripts in the selected RPDs. (**a**) The normalized reads per million mapped reads (NRPM) values of NM_199246 and NM_004060, two mRNA isoforms of the gene CCNG1, in the RPD (G1-1 synchronized Hela cells) are shown. (**b**) The translational level of NM_004060 is 2899.23. (**c**) The translational level of the gene CCNG1 is the sum of the ranslational levels of its two mRNA isoforms (NM_199246 and NM_004060).

Several software tools were used to process the ribo-seq reads (sra files) downloaded from GEO. First, SRAtoolkit v2.6.3 (https://www.ncbi.nlm.nih.gov/sra/docs/toolkitsoft/) was used to convert the sra files to fastq files. Second, Cutadapt v1.4.2 (31) was used to trim adaptor linker sequences or poly-(A) tails from the 3′ ends of reads. Third, RSEM (32) was used to align the reads to the reference human transcriptome and generate two files (the readdepth file and bam file). The reference human transcriptome (consisting of 38 401 mRNA transcripts) was downloaded from NCBI RefSeq database on 9 June 2017 (release 82). Fourth, SAMtools (33) was used to convert the bam file to sam file. Finally, our python script was used to calculate the total number of reads (which can be aligned to reference human transcriptome) from the sam file. The read counts of each coordinate of a reference human transcript (from the readdepth file) was then divided by a scaling factor (defined as the total number of mapped reads/1 000 000). The value of each coordinate of a reference human transcript now represents the normalized reads per million mapped reads (NRPM). The reason for doing the normalization is because the numbers of total mapped reads in different RPDs are different. We think that the normalization should be done before comparing the ribo-seq data mapped on the mRNAs from different RPDs. The NRPM values of each reference human transcript can then be plotted for visualization. For example, Figure 1a shows the NRPM values of NM_199246 and NM_004060 (two mRNA isoforms of the gene *CCNG1*) in the RPD: G1-1 synchronized Hela cells. For interested users, we have provided the detailed standard operating procedure and the bioinformatics scripts of the data analysis pipeline in the ‘Data processing’ section of the Help page.

### Calculation of translational levels of mRNA transcripts and genes

The translational level (*TL*) of a mRNA transcript in a RPD is defined as the average normalized reads per kilobase per million mapped reads (NRPKM) value of its coding region in that RPD and calculated by the following formula

TLmRNA=∑i=1LNRPMiL1000

where *L* is the length (in bps) of the coding region and *i* is the *i*-th position of the coding region. For example, in the RPD (G1-1 synchronized Hela cells), the *TL* of NM_004060 (one mRNA isoform of the gene *CCNG1*) is 2899.23 where *L *=* *887 (Figure 1b). Unlike RPKM/FPKM in RNA-seq which counts the reads on all exons, our translational level only counts the reads on CDS exons excluding 5′UTR exons and 3′UTR exons. To avoid misunderstanding, we did not use the term RPKM/FPKM.

Using RSEM (32,34), we define the translational level of a gene (denoted as ${TL}_{gene}$) in a RPD as the sum of the translational levels of all its mRNA isoforms in that RPD. RSEM treats mapping uncertainty in a statistically rigorous manner. RSEM uses a generative statistical model and associated inference methods that handle read mapping uncertainty in a principled manner (32,34). Since the isoform expression levels correspond to the model parameters, the individual isoform expression levels are directly estimated and the gene expression levels are estimated as the sum of estimated isoform expression levels (32,34). The authors of RSEM (34) demonstrated that the estimations of isoform expression levels and gene expression levels are more accurate than several existing methods. Note that the accuracy compared to other methods was assessed for RNA-seq and not Ribo-seq. Regarding RSEM’s assignment of footprint reads to two or more isoforms of the same gene, the confidence on correct footprint assignment depends on the uniqueness of the CDS regions of each isoform. The gene *CCNG1* has two mRNA isoforms (NM_199246 and NM_004060). In the RPD (G1-1 synchronized Hela cells), the ${TL}_{mRNA}$ of NM_199246 and ${TL}_{mRNA}$ of NM_004060 are 485.741 and 2899.23, respectively. Therefore, the ${TL}_{gene}$ of *CCNG1* is 3384.971 which equals the sum of 485.741 and 2899.23 (Figure 1c).

### Implementation of HRPDviewer website

HRPDviewer was built using the scripting language PHP and Codelgniter framework. All tables were produced by jQuery (a JavaScript library). All figures were generated using PHP GD library.

## Utility and discussion

### Database interface

**Figure 2.**
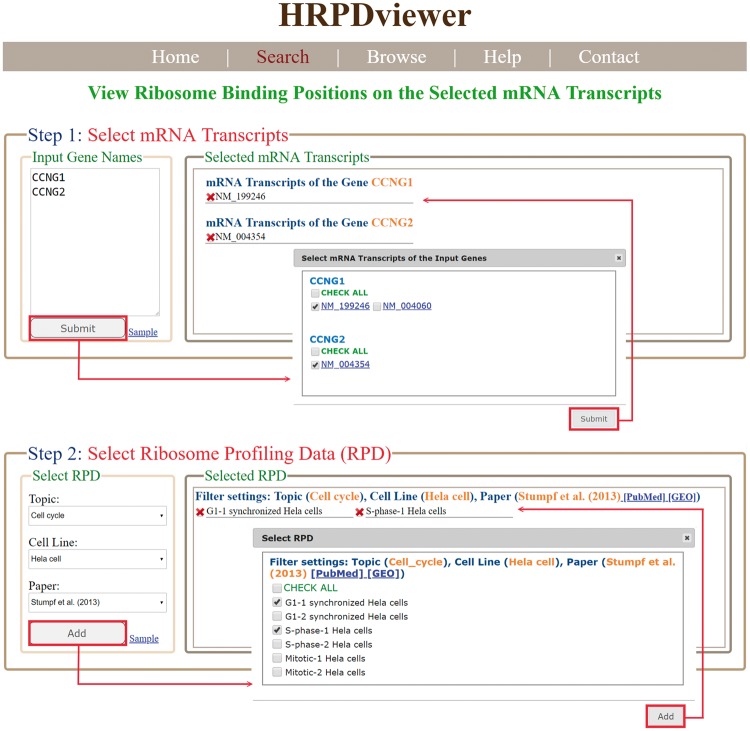
The input page of the search mode. Users have to select the mRNA transcripts and RPDs of interest.

**Figure 3.**
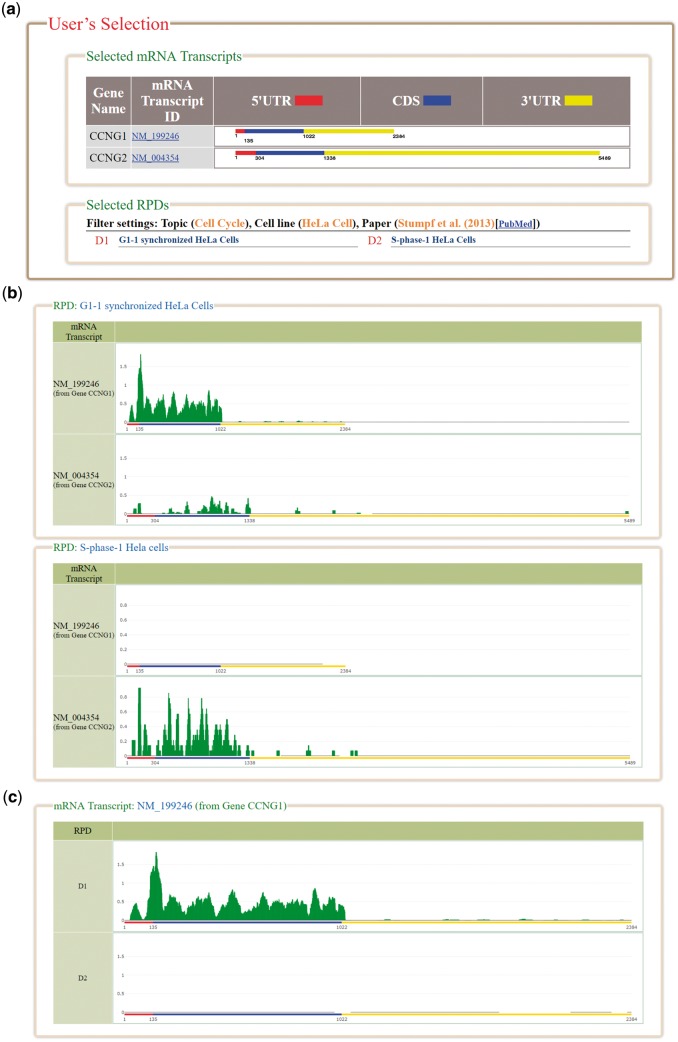
The result page of the search mode. (**a**) The information about the selected mRNA transcripts and RPDs is provided. (**b**) The ribosome binding patterns on different mRNA transcripts in the same RPD are shown. (**c**) The ribosome binding patterns on the same mRNA transcript in different RPDs are shown.

HRPDviewer provides both a search mode and a browse mode. In the search mode, users have to select the mRNA transcripts and RPDs of interest (Figure 2). After submission, HRPDviewer returns a result page containing two parts. The first part (Figure 3a) provides information about the selected mRNA transcripts and RPDs. The second part provides two different views of the ribosome binding patterns on the selected mRNA transcripts in the selected RPDs: (i) viewing different selected mRNA transcripts in the same RPD (Figure 3b) and (ii) viewing the same mRNA transcript in different RPDs (Figure 3c). The first view allows users to compare the ribosome binding patterns on different mRNA transcripts in the same RPD. Users then can know the translation of different mRNA transcripts under a specific physiological condition. For example, NM_199246 (one mRNA isoform of the gene *CCNG1*) is more actively translated than NM_004354 (the only one mRNA isoform of the gene *CCNG2*) in G1 synchronized Hela cells. On the contrary, NM_004354 is more actively translated than NM_199246 in S phase synchronized Hela cells (Figure 3b). Note that NM_004060 (the other one mRNA isoform of the gene *CCNG1*) is more actively translated than both NM_199246 and NM_004354 in both G1 and S phase (data not shown). The second view allows users to compare the ribosome binding patterns on an mRNA transcript in different RPDs. Users then can know the translation of a specific mRNA transcript under different physiological conditions. For example, NM_199246 (one mRNA isoform of the gene *CCNG1*) is more actively translated in G1 phase than in S phase of the cell cycle in Hela cells (Figure 3c). Note that all the ribo-seq data of the G1 and S phases of the cell cycle come from Stumpf *et al.* (35) who used ribo-seq technology to investigate the translational landscape of the mammalian cell cycle.

**Figure 4.**
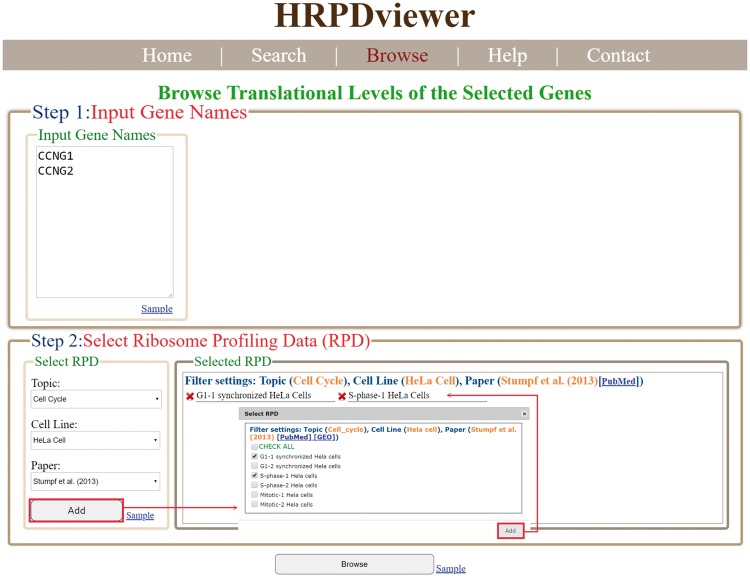
The input page of the browse mode. Users have to input a list of genes and select RPDs to be shown.

**Figure 5.**
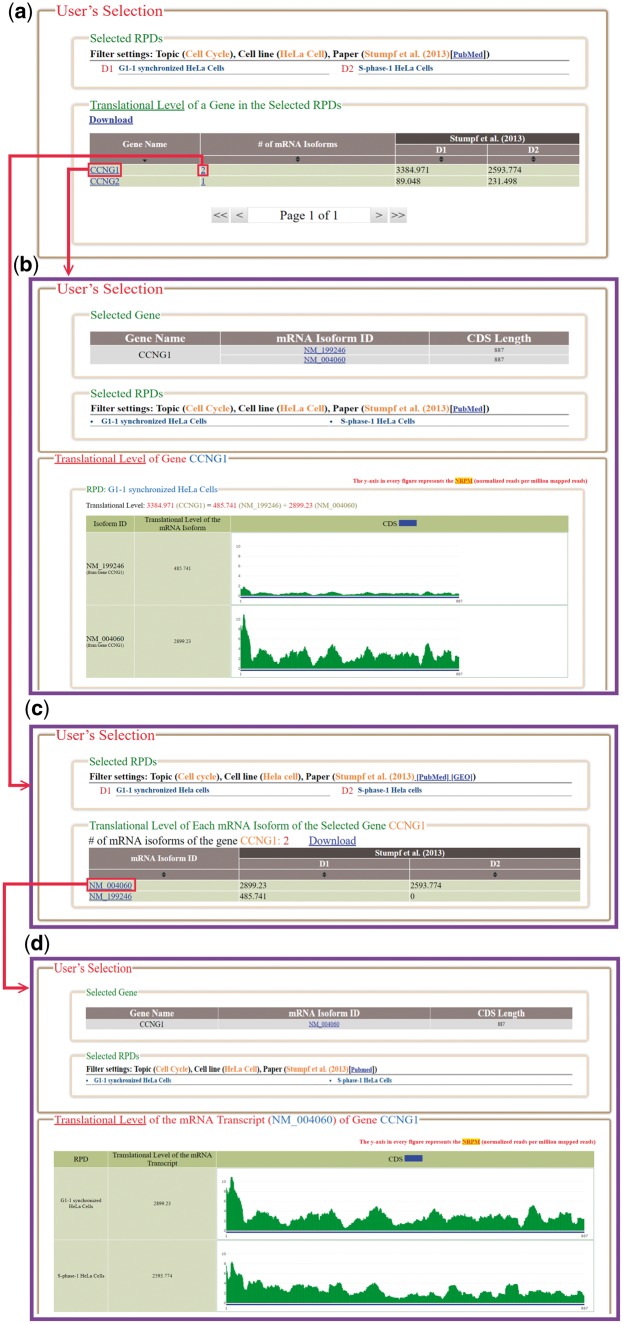
The result page of the browse mode. (**a**) The information (gene name, the number of mRNA isoforms and the translational levels in the selected RPDs) of each gene to be shown is provided. (**b**) When clicking on an item in the ‘Gene Name’ (e.g. CCNG1), HRPDviewer returns a page showing how the translational levels of the gene CCNG1 in different RPDs are calculated. (**c**) When clicking on an item in the ‘# of mRNA Isoforms’, HRPDviewer returns a page containing information (mRNA isoform ID and the translational level in the selected RPDs) of each mRNA isoform of the selected gene. (**d**) When clicking on item in the ‘isoform ID’ (e.g. NM_004060), HRPDviewer returns a page showing how the translational levels of NM_004060 in different RPDs are calculated.

In the browse mode, users have to (i) input a list of genes of interest and (ii) select RPDs to be shown (Figure 4). After submission, HRPDviewer returns a page containing information (gene name, the number of mRNA isoforms and the translational levels in the selected RPDs) of each gene to be shown (Figure 5a). When clicking on an item in the ‘Gene Name’ (e.g. *CCNG1*), HRPDviewer returns a page showing the translational levels of all the isoforms of the gene *CCNG1* in the selected RPDs (Figure 5b). When clicking on an item in the ‘# of mRNA Isoforms’, HRPDviewer returns a page containing information (mRNA isoform ID and the translational level in the selected RPDs) of each mRNA isoform of the selected gene (Figure 5c). When clicking an item in the ‘isoform ID’ (e.g. NM_004060), HRPDviewer returns a page showing the NRPM values on each nucleotide position in the CDS of NM_004060 in the selected RPDs (Figure 5d).

**Figure 6.**
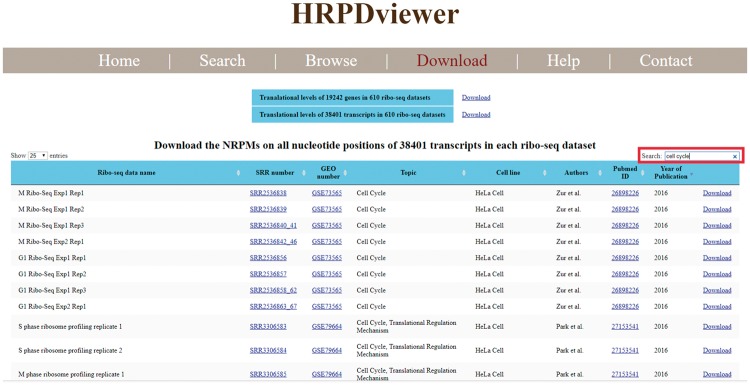
Download page. In the download page, users can download the translational levels of all genes and all transcripts in each of the 610 RPDs. The NRPM values on all nucleotide positions of all transcripts in each of 610 RPDs can also be downloaded. Using the keyword search (e.g. cell cycle), users can sub-select some ribo-seq datasets suitable for their plans or research. For each ribo-seq data, we have provided links to Sequence Read Archive (SRA), Gene Expression Omnibus (GEO) and Pubmed.

HRPDviewer allows users to download all the figures and the corresponding raw data in the search/browse result page. Moreover, HRPDviewer provides a download page for users to download the translational levels of all genes and all transcripts in each of the 610 RPDs (Figure 6). The NRPM values on all nucleotide positions of all transcripts in each of 610 RPDs can also be downloaded. Using the keyword search (e.g. cell cycle), users can sub-select some ribo-seq datasets suitable for their plans or research. For each ribo-seq data, we have provided links to Sequence Read Archive (SRA), GEO, and Pubmed. For each gene and transcript, we have provided links to NCBI.

### A case study

Transitions of the eukaryotic cell cycle phases are governed by cyclin-dependent kinases whose activities are regulated by cyclins. Mammals have a family of eight cyclins (cyclins A through H). Two cyclin G proteins (G1 and G2) play roles during the DNA damage response and their dysfunctions lead to breast/lung/gastric cancers (36–40). Cyclin G1 (encoded by the gene *CCNG1*) lacks a protein destabilizing (PEST) motif that is present in other family members and two mRNA isoforms (NM_004060 and NM_199246) of *CCNG1* have been identified. Cyclin G2 (encoded by the gene *CCNG2*) is the cyclin G1 homolog with 53% amino acid sequence identity. Unlike cyclin G1, cyclin G2 contains a PEST motif, indicating that cyclin G2 expression might be tightly cell-cycle regulated. Indeed, it has been shown that, in human lymphocytes, cyclin G1 mRNA is constitutively expressed throughout the cell cycle with peak levels at early G1 phase but cyclin G2 mRNA level exhibits cell cycle periodicity with peak levels at mid-S phase (41).

**Figure 7.**
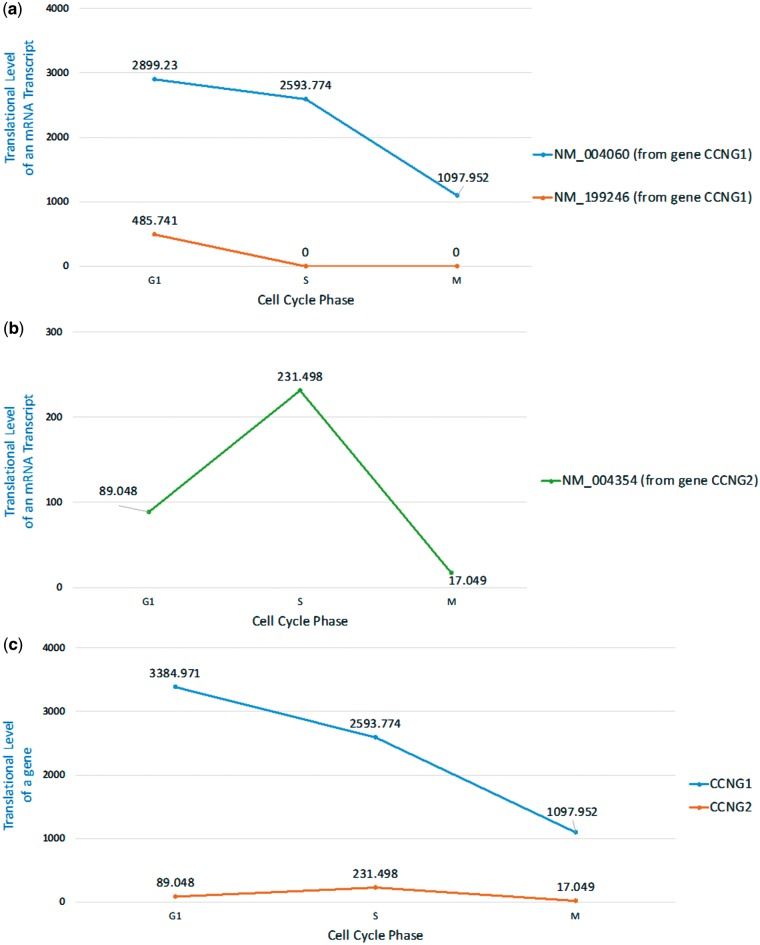
Translational levels of the selected mRNA transcripts and genes in three selected cell cycle RPDs (G1 phase, S phase and M phase). (**a**) The translational levels of NM_004060 and NM_199246 (the two mRNA isoforms of the gene CCNG1) are shown. (**b**) The translational levels of NM_004354 (the mRNA transcript of the gene CCNG2) are shown. (**c**) The translational levels of the genes CCNG1 and CCNG2 are shown.

Here we are interested in checking whether we can see the same phenomenon of cyclin G1 and cyclin G2 at the translational level. To do that, we looked at the ribo-seq data from a mammalian cell cycle study in Hela cell (35). Using our HRPDviewer, we were able to see that the cyclin G1 mRNA isoform NM_004060 is constitutively translated throughout the cell cycle with peak levels at early G1 phase but the other cyclin G1 mRNA isoform NM_199246 is only translated in G1 phase (Figure 7a and Supplementary Figure S1). However, since the gene *CCNG1*’s two isoforms (NM_004060 and NM_199246) differ only in the 5′UTR exons, whether NM_199246 is really only translated in G1 phase needs further experimental validation (Supplementary File S1). On the other hand, the translation of cyclin G2 mRNA transcript NM_004354 exhibits cell cycle periodicity with peak levels at mid-S phase (Figure 7b and Supplementary Figure S2). Moreover, the translational level of *CCNG1* (the gene which encodes cyclin G1) is much higher than that of *CCNG2* (the gene which encodes cyclin G2) (Figure 7c and Supplementary Figures S1 and S2). In summary, our HRPDviewer helps users easily know that (i) the two mRNA isoforms of the gene *CCNG1* may be under different translational regulations and (ii) the two genes *CCNG1* and *CCNG2* are under different translational regulations.

## Discussion

In an exploratory study, users can download the translational levels of all transcripts/genes in two conditions [e.g. G1 phase and S phase of the cell cycle (35)] from the download page of HRPDviewer. Then users themselves can extract the transcripts/genes whose translational levels change significantly (e.g. fold change ≥2) for further investigation.

Some early ribo-seq studies (e.g. GSE93133, GSE59815 and GSE59816) did not generate any RNA-seq data. Some other studies (e.g. GSE80156 and GSE87328) generated ribo-seq data in many conditions but RNA-seq in fewer conditions. Since not every Ribo-seq data has a corresponding RNA-seq data, we do not include RNA-seq data in this version of HRPDviewer. However, we will include RNA-seq data in our future update.

Since our ribo-seq data pipeline can be applied to other species, we will include the ribo-seq data of other species in our future update. We limit ourselves to Human in this first version of HRPDviewer because it takes a lot of time to collect and process the published ribo-seq datasets of all species. We think that Human is the most important species, so at this stage we construct the database just for Human. We will search GEO database every 6 months for new published ribo-seq data. Once we find new data, we will process and include the data in our database. We will also keep up with changes of transcriptome or genome annotations every 6 months.

## Conclusion

In this study, we developed HRPDviewer. HRPDviewer (i) collected 610 published human ribo-seq datasets from GEO, (ii) aligned the ribo-seq data to the transcriptome and (iii) provided visualization of the ribo-seq data on the selected mRNA transcripts. Users can compare and visualize the ribo-seq data mapped on different mRNA transcripts under different physiological conditions. These visualizations enable users to see not only which genes’ mRNA transcripts are highly translated, but also which mRNA isoforms of the same gene are highly translated under a specific physiological condition. This kind of visualization provides novel biological insights, as illustrated by the included case study. We believe that HRPDviewer is a useful resource for studying translational regulation in human.

## Supplementary data

Supplementary data are available at Database online.

## Supplementary Material

Supplementary Data
