# Supplementary material for: HRPDviewer: human ribosome profiling data viewer
Source: Database (Oxford). 2018 Jul 11;2018:bay074. doi: 10.1093/database/bay074 (PMC6041748; doi:10.1093/database/bay074)
Supplement: Supplementary Data [file bay074_supp.zip › Supplementary File 1.pdf]

Supplementary File 1

Supplementary Figure 1 shows that the calculated translational level for the S-phase and M-phase for the NM_004060 isoform of the CCNG1 gene is relatively high while zero for the other NM_199246 isoform of CCNG1. Yet the two isoforms of the CCNG1 gene differ in the 5' leader exonic architecture while sharing the same CDS sequence composition (they have the same CCDS annotation CCDS4360.1).

RSEM was designed for RNA-seq but not for Ribo-seq. As Ribo-seq footprints primarily originate from CDS regions, accurate attribution of footprints to a particular isoform over others would rely on that isoform's unique differences in CDS exonic sequence composition. Unlike with RNA-seq, differences in 5' leader and 3' trailer sequence composition cannot be relied upon for mapping statistics with Ribo-seq since it cannot be assumed that footprints will originate from regions outside of the CDS.

Q: In other words, footprints in a 5' leader region may help delineate the isoform which is being translated, but what happens if the density of footprints in the 5' leader region is very low or absent? Hence, can the authors comment on the accuracy of RSEM for individual isoform ribosome footprint attribution?

A: RSEM treats mapping uncertainty in a statistically rigorous manner. RSEM uses a generative statistical model and associated inference methods that handle read mapping uncertainty in a principled manner. Since the isoform expression levels correspond to the model parameters, the individual isoform expression levels are directly estimated and the gene expression levels are estimated as the sum of estimated isoform expression levels. The authors of RSEM demonstrated that the estimations of isoform expression levels and gene expression levels are more accurate than several existing methods.

The reason that, in S-phase, RSEM redistributes almost all mapped reads to NM_004060 isoform and almost none to NM_199246 is because there is **a relatively high number of unique reads** mapped to the unique region of NM_004060 but **no unique reads (only mutli-reads)** mapped to the unique region of NM_199246 (Appendix 1). Therefore, the redistribution of mapped reads among isoforms by RSEM seems methodologically reasonable. However, we agree with the reviewer’s concern. Since the unique regions of these two isoforms are in the 5’UTR exons, the low density of footprints in the unique region of NM_199246 may not necessarily mean that NM_199246 is not being

actively translated. Nevertheless, in other cases, if the differences of two isoforms are in the CDS exons (e.g. the two isoforms NM_001142604 and NM_00310 of PPT1, Appendix 2), the results of RSEM should be trustworthy. Fortunately, **most (87%=33546/38401) isoforms differ in CDS exons** because we found 33546 CCDS annotations among 38401 mRNA transcripts. Therefore, the accuracy of RSEM for individual isoform ribosome footprint attribution should be acceptable.

Appendix 1

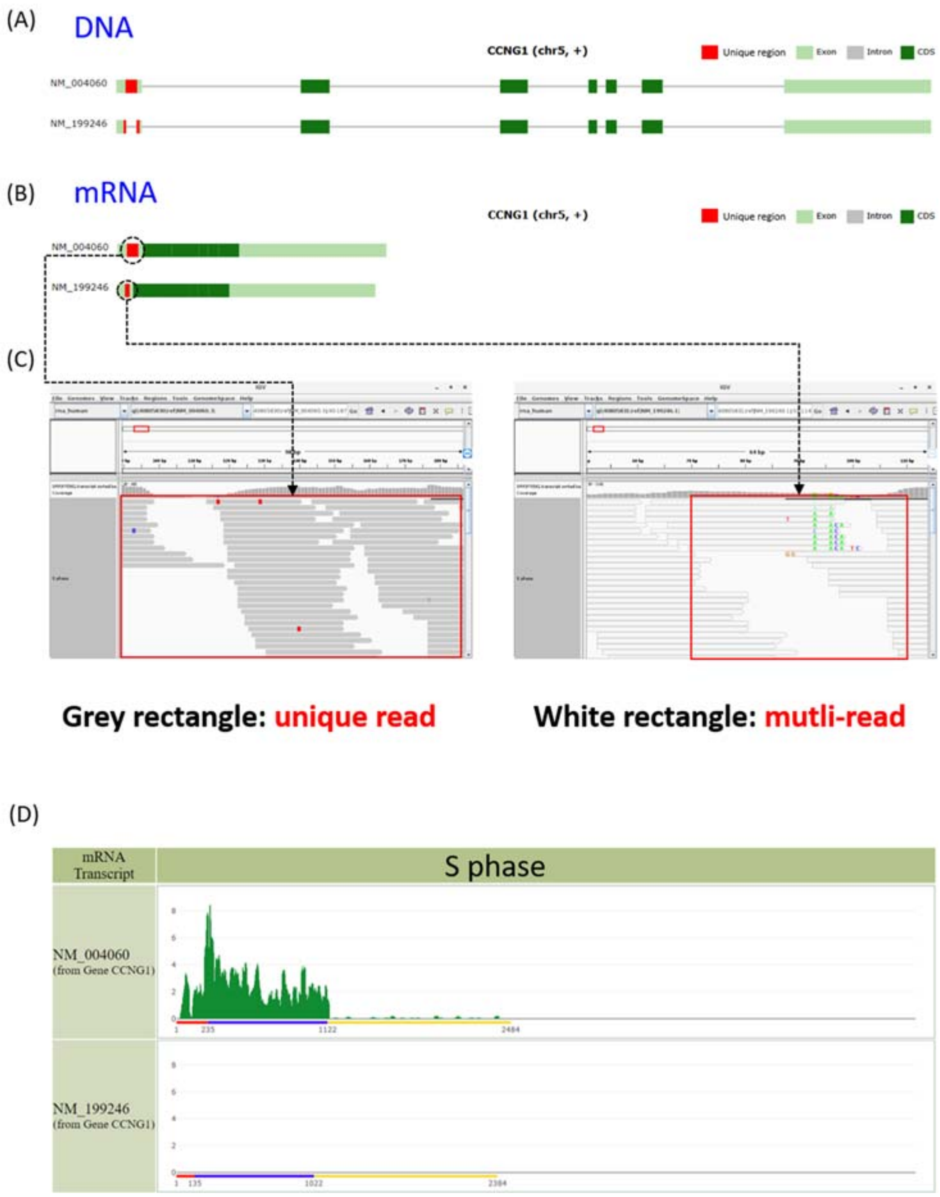

The reason that, in S-phase, RSEM redistributes almost **all mapped reads (from Bowtie)** to NM_004060 isoform and almost none to NM_199246 is because there is **a relatively high number of unique reads (grey rectangles)** mapped to the **unique region of NM_004060** but **no unique reads (only multi-reads (white rectangles))** mapped to the **unique region of NM_199246**. Therefore, the redistribution of mapped reads among isoforms by RSEM seems methodologically reasonable. However, since the unique regions of these two isoforms are in the 5'UTR exons, the

low density of footprints in the unique region of NM_199246 may not necessarily mean that NM_199246 is not being actively translated.

Appendix 2

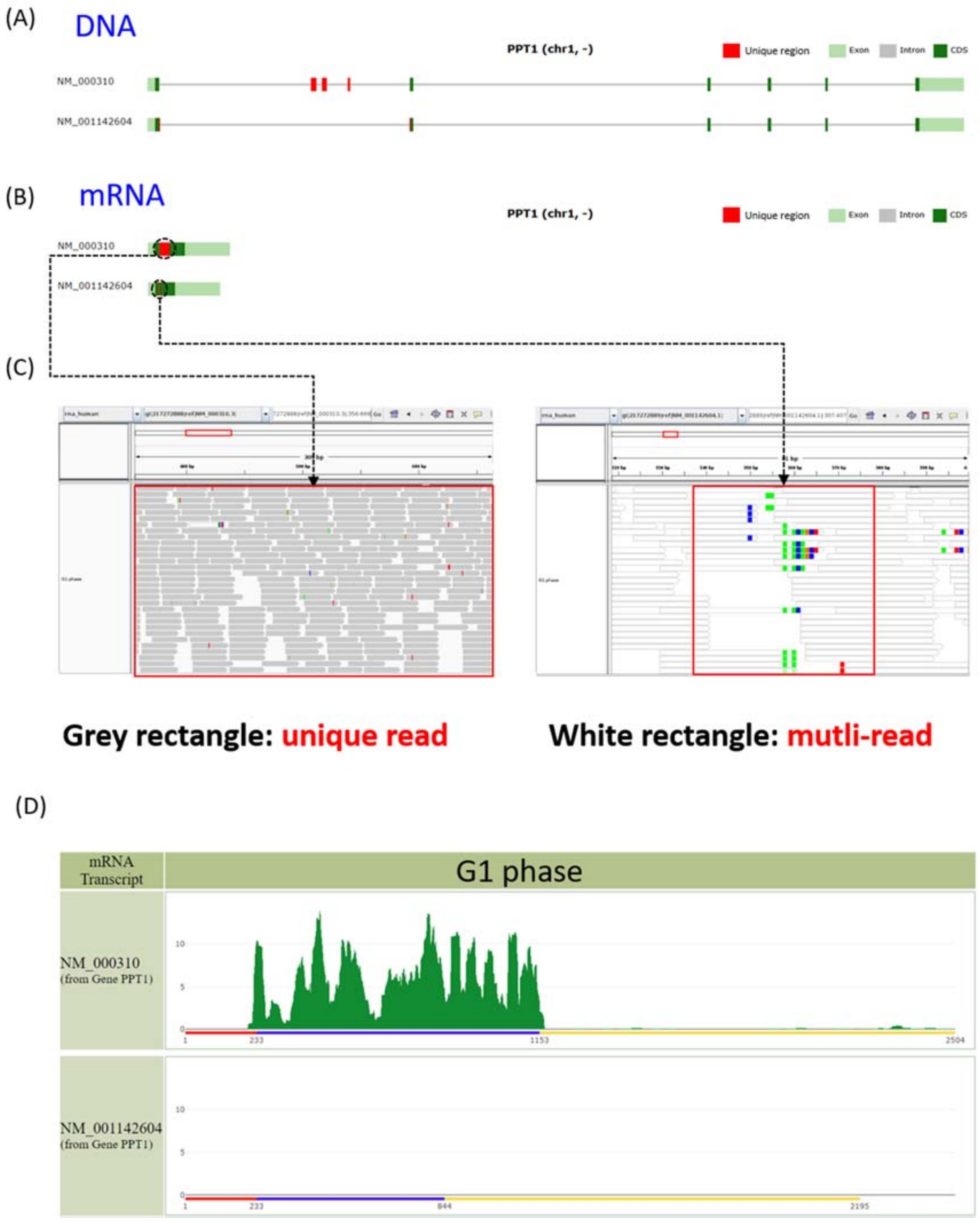

In other cases, if the differences of two isoforms are in the CDS exons (e.g. the two isoform NM_00310 and NM_00142604 of PPT1), the results of RSEM should be

trustworthy.
