# Supplementary material for: HRPDviewer: human ribosome profiling data viewer
Source: Database (Oxford). 2018 Jul 11;2018:bay074. doi: 10.1093/database/bay074 (PMC6041748; doi:10.1093/database/bay074)
Supplement: Supplementary Data [file bay074_supp.zip › Supplementary Figure 1.pdf]

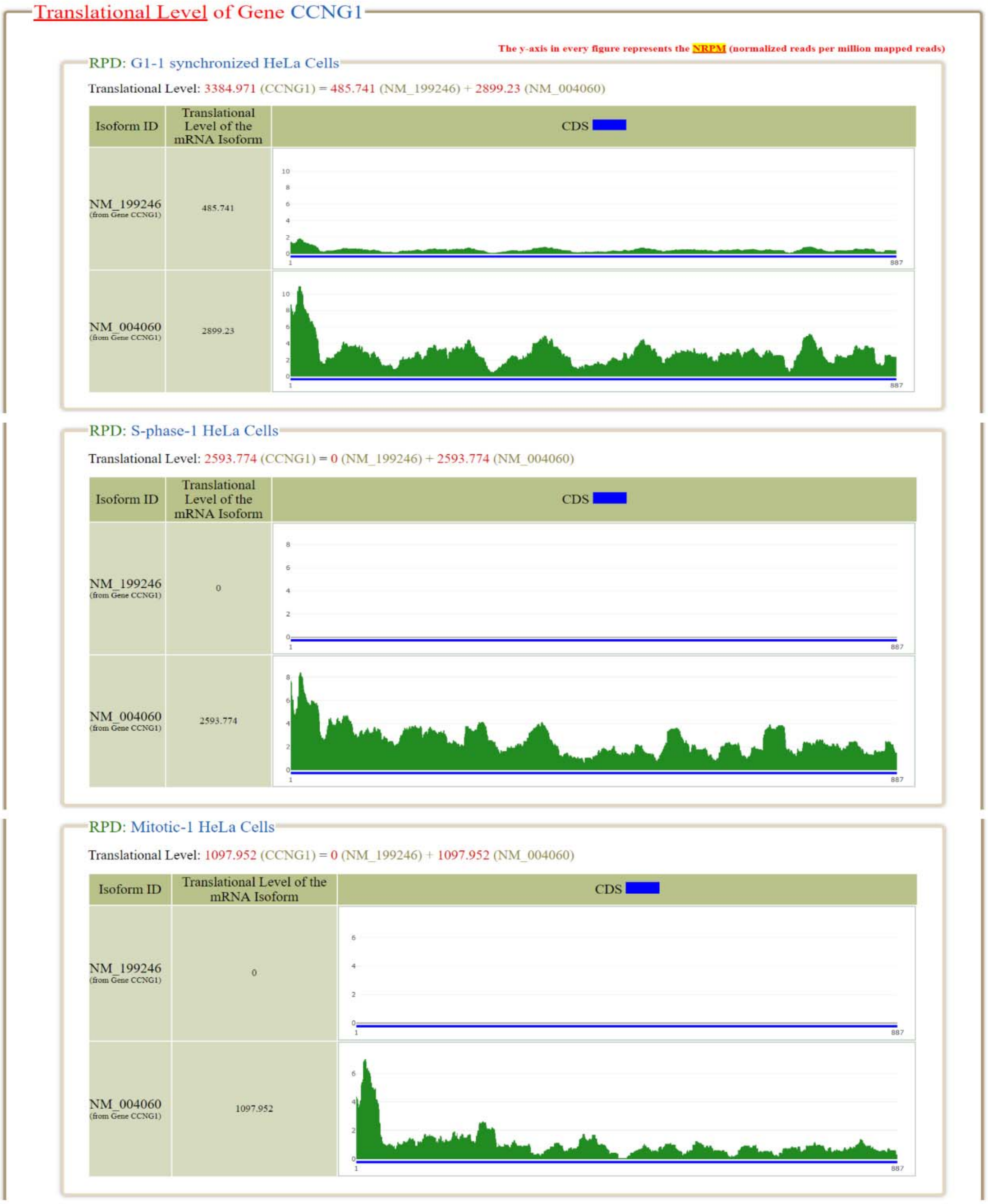

Supplementary Figure 1
